# Artificial Noise Injection and Its Power Loading Methods for Secure Space-Time Line Coded Systems

**DOI:** 10.3390/e21050515

**Published:** 2019-05-22

**Authors:** Jingon Joung, Jihoon Choi, Bang Chul Jung, Sungwook Yu

**Affiliations:** 1 School of Electrical and Electronics Engineering, Chung-Ang University, Seoul 06974, Korea; 2 School of Electronics and Information Engineering, Korea Aerospace University, Gyeonggi-do 10540, Korea; 3 Department of Electronics Engineering, Chungnam National University, Daejeon 34134, Korea

**Keywords:** space-time line code, physical layer security, secrecy rate, artificial noise, power control

## Abstract

In this paper, we consider a 2×2 space-time line coded (STLC) system having two-transmit and two-receive antennas. To improve the secrecy rate of the STLC system, in which an illegitimate receiver eavesdrops the information delivered from the STLC transmitter to the STLC receiver, we propose an artificial noise (AN) injection method. By exploiting the STLC structure, a novel AN for the STLC is designed and its optimal power loading factor is derived. Numerical results verify that the proposed secure STLC systems with the designed AN injection and the power control method can significantly improve the secrecy rate compared to the conventional STLC systems. It is observed that the proposed method is more effective if there is a significant gap between the main-channel and the eavesdropper-channel gains.

## 1. Introduction

For secure wireless communications, along with cryptographic encryption on an application layer, physical-layer security (PLS) technologies [1,2,3] have been attracting intensive research interest from various fields with numerous successful applications, such as wireless power transmission systems [4,5], massive multi-input multi-output (MIMO) systems [6,7], millimeter wave systems [8], and unmanned aerial vehicle systems [9,10,11,12]. Contrary to an anti-jamming scheme that intends to remove the jamming signals from the received signals [13,14], i.e., the data protection from the jammer’s attack, the PLS technologies are mainly focused on the data protection from being eavesdropped by the eavesdropper. Various practical PLS techniques, such as the precoding/beamforming schemes [15,16], the cooperation methods [17,18,19,20], the secrecy-achieving codes [21,22,23], and artificial noise (AN) injection [24,25,26,27,28,29], have been studied. Among these, the AN injection methods have been vigorously studied due to their simplicity and effectiveness. By adding AN to the transmitted signals at a transmitter (i.e., Alice), the secrecy capacity can be significantly improved as the AN affects as an interference only on an eavesdropper (i.e., Eve), not a legitimate user (i.e., Bob). In [27,28], an AN was designed for a secure space-time block coded (STBC) system to improve the secrecy rate of the STBC systems.

In this paper, we consider a two-antenna secure system, in which all devices including Alice, Bob, and Eve have two antennas. Following a vast majority of studies (see [30] and the references therein), both the main and the eavesdropper channels are assumed to be available at Alice. Nevertheless, Eve’s channel cannot be nullified due to the lack of spatial degrees of freedom. Furthermore, no nullspace of the main (Bob’s) channel exists without any receive-combining technique. Thus, we employed a space-time line code (STLC) scheme in [31], which is a dual transmission scheme of space-time block code [32]. The STLC scheme has been applied to various communication systems, e.g., the multiuser systems [33,34], two-way relay systems [35,36], antenna shuffling systems [37], and blind decoding systems [38]. Here, channel state information (CSI) is assumed to be available at the transmitter under the assumption that the uplink and downlink channels are symmetric in time-division duplex (TDD) mode. Assuming the TDD mode in a two-antenna secure system considered in this paper, the channels from Bob to Alice are symmetric with the channels from Alice to Bob. Hence, the legitimate downlink channels (Bob’s channel) can be obtained by estimating the uplink channels at Alice. Since STLC can achieve full spatial diversity gain with a single transmit antenna, the other transmit antenna can be utilized to generate AN. The AN is designed such that it is perfectly canceled out after decoding the STLC symbols at Bob, and its optimal power loading factor is then designed to maximize the secrecy rate, i.e., a sort of resource management scheme [34,36,39]. Furthermore, the STLC schemes can be implemented with full-blind (non-coherent) detection at the legitimate receiver [38], and the transmitter does not need to transmit long-training sequences, which also improve the secrecy rate of the network as this hinders the eavesdroppers from estimating their own channels. Numerical results verify that the designed power loading strategy maximizes the secrecy rate, and the proposed secure STLC with the power controlled AN can significantly improve PLS of the 2×2 STLC systems.

Notations: Superscripts *T* and * denote transposition and complex conjugate, respectively, for any scalar, vector, or matrix. The notations |x| and ∥x∥ denote the absolute value of *x* and the norm of a vector x, respectively; null(X) gives the span of the nullspace of X; and $x∼\mathrm{CN}(0,\sigma^{2})$ means that a complex random variable *x* conforms to a normal distribution with a zero mean and variance $\sigma^{2}$. E[x] stands for the expectation of a random variable *x*.

## 2. Proposed 2×2 Secure STLC with AN

**Figure 1:**
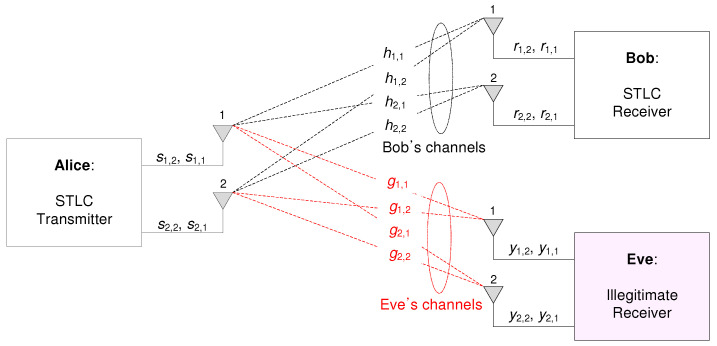
The 2×2 secure space-time line coded (STLC) system model, with a transmitter (Alice) and legitimate receiver (Bob). Here, one eavesdropper (Eve) eavesdrops actively.

Consider an STLC system as shown in Figure 1, in which Alice sends two information symbols, $x_{1}$ and $x_{2}$, through two consecutive transmissions to Bob. Here, Eve eavesdrops the information. All devices are assumed to have two antennas, i.e., two 2×2 STLC systems, and without loss of generality, $E[|x{|}^{2}]=1$. Let $s_{m,t}$ be an STLC symbol that is transmitted through the $m^{\mathrm{th}}$ transmit antenna at time *t*, where m,t∈{1,2}. Denote AN by z∼CN(0,1) and its complex-value weight by $a_{m,t}$ such that:

(1) $$E\left[|a_{1,1}{|}^{2}+|a_{2,1}{|}^{2}+|a_{1,2}{|}^{2}+{\left|a_{2,2}\right|}^{2}\right]=2.$$

The proposed secure STLC symbols with AN are then written as follows:

(2a) $$\begin{array}{ccc} s_{1,1} & = & \sqrt{1-\alpha }\sqrt{\frac{1}{\gamma }}\left(h_{1,1}^{*}x_{1}+h_{2,1}^{*}x_{2}^{*}\right)+\sqrt{\alpha }a_{1,1}z, \end{array}$$

(2b) $$\begin{array}{ccc} s_{1,2} & = & \sqrt{1-\alpha }\sqrt{\frac{1}{\gamma }}\left(h_{2,1}^{*}x_{1}^{*}-h_{1,1}^{*}x_{2}\right)+\sqrt{\alpha }a_{1,2}z^{*}, \end{array}$$

(2c) $$\begin{array}{ccc} s_{2,1} & = & \sqrt{1-\alpha }\sqrt{\frac{1}{\gamma }}\left(h_{1,2}^{*}x_{1}+h_{2,2}^{*}x_{2}^{*}\right)+\sqrt{\alpha }a_{2,1}z, \end{array}$$

(2d) $$\begin{array}{ccc} s_{2,2} & = & \sqrt{1-\alpha }\sqrt{\frac{1}{\gamma }}\left(h_{2,2}^{*}x_{1}^{*}-h_{1,2}^{*}x_{2}\right)+\sqrt{\alpha }a_{2,2}z^{*}, \end{array}$$

where $h_{n,m}∼\mathrm{CN}\left(0,1\right)$ represents the channel gain from the $m^{\mathrm{th}}$ transmit antenna of Alice to the $n^{\mathrm{th}}$ receive antenna of Bob;

(3) $$\gamma =|h_{1,1}{|}^{2}+|h_{2,1}{|}^{2}+|h_{1,2}{|}^{2}+{\left|h_{2,2}\right|}^{2}$$

is for the transmit power normalization such that:

(4) $$E\left[|s_{1,1}{|}^{2}+|s_{2,1}{|}^{2}+|s_{1,2}{|}^{2}+{\left|s_{2,2}\right|}^{2}\right]=2;$$

and α is the power loading factor for the AN signals, where 0≤α≤1. According to the AN-power loading factor, α, three operation modes can be considered as follows:

- α=0: a conventional STLC mode without AN [31,33],
- 0<α<1: a secure STLC mode with AN,
- α=1: a jamming mode without data transmission.

Herein, we focus on the secure STLC mode in this letter.

Denoting $r_{n,t}$ as the received signal at antenna *n* of Bob at time *t*, the four received signals are then written as follows (n,t∈{1,2}):

(5) $$r_{n,t}=h_{n,1}s_{1,t}+h_{n,2}s_{2,t}+v_{n,t},$$

where $v_{n,t}∼\mathrm{CN}\left(0,\sigma^{2}\right)$ is the additive white Gaussian noise at $r_{n,t}$.

The received symbols $r_{1,1}$ and $r_{2,2}$ in Equation (5) are directly combined to obtain the estimate of $x_{1}$ and $x_{2}$, which are derived by using Equations (2)–(5) as follows:

(6a)x˜1=r1,1+r2,2*(6b)=h1,1s1,1+h1,2s2,1+v1,1+h2,1*s1,2*+h2,2*s2,2*+v2,2*=h1,11−α1γh1,1*x1+h2,1*x2*+αa1,1z+h1,21−α1γh1,2*x1+h2,2*x2*+αa2,1z+h2,1*1−α1γh2,1*x1*−h1,1*x2+αa1,2z**(6c)+h2,2*1−α1γh2,2*x1*−h1,2*x2+αa2,2z**+v1,1+v2,2*=1−α1γ|h1,1|2x1+h1,1h2,1*x2*+|h1,2|2x1+h1,2h2,2*x2*+αh1,1a1,1+h1,2a2,1z+1−α1γ|h2,1|2x1−h1,1h2,1*x2*+|h2,2|2x1−h1,2h2,2*x2*+αh2,1*a1,2*+h2,2*a2,2*z(6d)+v1,1+v2,2*(6e)=1−α1γγx1+αh1,1a1,1+h1,2a2,1+h2,1*a1,2*+h2,2*a2,2*z+v1,(6f)=1−αγx1+αh1,1a1,1+h1,2a2,1+h2,1*a1,2*+h2,2*a2,2*z+v1,

where the first, second, and third terms of the right-hand side (RHS) of Equation (6f) are the intended signal, the interferences caused by AN, and the combined noise $v_{1}=v_{1,1}+v_{2,2}^{*}∼\mathrm{CN}\left(0,2\sigma^{2}\right)$, respectively.

Similarly, combining $r_{1,2}$ and $r_{2,1}$ in Equation (5), the estimate of $x_{2}$ is obtained as follows:

(7a)x˜2=r2,1*−r1,2(7b)=h2,1*s1,1*+h2,2*s2,1*+v2,1*−h1,1s1,2−h1,2s2,2−v1,2=h2,1*1−α1γh1,1*x1+h2,1*x2*+αa1,1z*+h2,2*1−α1γh1,2*x1+h2,2*x2*+αa2,1z*−h1,11−α1γh2,1*x1*−h1,1*x2+αa1,2z*(7c)−h1,21−α1γh2,2*x1*−h1,2*x2+αa2,2z*+v2,1*−v1,2=1−α1γh2,1*h1,1x1*+|h2,1|2x2+h2,2*h1,2x1*+|h2,2|2x2+αh2,1*a1,1*+h2,2*a2,1*z*+1−α1γ−h1,1h2,1*x1*+|h1,1|2x2−h1,2h2,2*x1*+|h1,2|2x2−αh1,1a1,2+h1,2a2,2z*(7d)+v1,1+v2,2*(7e)=1−α1γγx2+αh2,1*a1,1*+h2,2*a2,1*−h1,1a1,2−h1,2a2,2z*+v2,(7f)=1−αγx2+αh2,1*a1,1*+h2,2*a2,1*−h1,1a1,2−h1,2a2,2z*+v2,

where the first, second, and third terms of the RHS of Equation (7f) are the intended signal, the interferences caused by AN, and the combined noise $v_{2}=v_{2,1}^{*}-v_{1,2}∼\mathrm{CN}\left(0,2\sigma^{2}\right)$.

To eliminate the AN effects on Bob perfectly, the second terms in the RHS of Equations (6f) and (7f) should be a zero as follows:

(8a) $$\begin{array}{ccc} h_{1,1}a_{1,1}+h_{1,2}a_{2,1}+h_{2,1}^{*}a_{1,2}^{*}+h_{2,2}^{*}a_{2,2}^{*} & = & 0 \end{array}$$

(8b) $$\begin{array}{ccc} h_{2,1}^{*}a_{1,1}^{*}+h_{2,2}^{*}a_{2,1}^{*}-h_{1,1}a_{1,2}-h_{1,2}a_{2,2} & = & 0. \end{array}$$

Therefore, the AN weights, $\left\{a_{m,t}\right\}$, should fulfill the following conditions, which are a matrix and vector representation of Equation (8):

(9) $$\left[\begin{array}{cccc} h_{1,1} & h_{2,1}^{*} & h_{1,2} & h_{2,2}^{*}\\ h_{2,1} & -h_{1,1}^{*} & h_{2,2} & -h_{1,2}^{*} \end{array}\right]\left[\begin{array}{c} a_{1,1}\\ a_{1,2}^{*}\\ a_{2,1}\\ a_{2,2}^{*} \end{array}\right]=Ha=\left[\begin{array}{c} 0\\ 0 \end{array}\right].$$

From Equation (9), the AN weights are obtained as follows:

(10) $$\begin{array}{c} a=\sqrt{2}\;\mathrm{null}\;\left(H\right)\in C^{4\times 1}, \end{array}$$

where null(H) gives the span of the nullspace of a matrix H, which is a 4×1 unit-norm vector, and thus, $|a_{1,1}{|}^{2}+|a_{2,1}{|}^{2}+|a_{1,2}{|}^{2}+{\left|a_{2,2}\right|}^{2}=2$. Using Equations (6f), (7f) and (10), the combined STLC signals turn into the interference-free estimates as:

(11) $${\tilde{x}}_{i}=\sqrt{1-\alpha }\sqrt{\gamma }\;x_{i}+v_{i},\;i\in \left\{1,\;2\right\}.$$

Noting that $x_{1}$ and $x_{2}$ are not coupled with each other in Equation (11), two separate maximum-likelihood detections can be applied to detect $x_{1}$ and $x_{2}$ independently. Here, the detection signal-to-noise (SNR) is readily derived as:

(12) $$\rho_{\mathrm{Bob}}=\frac{(1-\alpha )\gamma }{2\sigma^{2}}.$$

From Equation (12), we verify that the secure STLC achieves performance identical to that of the conventional STLC and STBC in terms of the spatial diversity gain.

As we extend the number of transmit antennas from two to *M*, where *M* is an even number for simple derivation, which is not a necessary condition of *M* in practice, the AN effects on Bob Equation (8) can be generally written as follows:

(13a) $$\begin{array}{ccc} \sum_{m=1}^{M}\left(h_{1,m}a_{1,m}+h_{2,m}^{*}a_{2,m}^{*}\right) & = & 0 \end{array}$$

(13b) $$\begin{array}{ccc} \sum_{m=1}^{M}\left(h_{2,m}^{*}a_{1,m}^{*}-h_{1,m}a_{2,m}\right) & = & 0. \end{array}$$

Thus, the AN weights, $\left\{a_{m,t}\right\}$, should fulfill the following conditions:

(14) $$\left[\begin{array}{cccccccc} h_{1,1} & h_{2,1}^{*} & \cdots & h_{1,m} & h_{2,m}^{*} & \cdots & h_{1,M} & h_{2,M}^{*}\\ h_{2,1} & -h_{1,1}^{*} & \cdots & h_{2,m} & -h_{1,m}^{*} & \cdots & h_{2,M} & -h_{1,M}^{*} \end{array}\right]a=Ha=\left[\begin{array}{c} 0\\ 0 \end{array}\right],$$

where:

(15) $$a={\left[\begin{array}{cccccccc} a_{1,1} & a_{1,2}^{*} & \cdots & a_{2,m} & a_{2,m}^{*} & \cdots & a_{1,M} & a_{1,M}^{*} \end{array}\right]}^{T}.$$

From this, the AN weights are obtained as follows:

(16) $$\begin{array}{c} a=\sqrt{M}\;\mathrm{null}\;\left(H\right)\in C^{2M\times 1}. \end{array}$$

The computational complexity to design AN for *M* transmit antenna STLC systems, i.e., M×2 STLC systems, is roughly $O\left({\left(2M\right)}^{3}\right)=O\left(M^{3}\right)$. Since the computational complexity to design a power control factor α, which is shown in the next section, is lower than that for the AN design, the total computational complexity of the proposed method is $O(M^{3})$.

## 3. Power Control for AN

Denote Eve’s channel gain between the $m^{\mathrm{th}}$ transmit antenna of Alice and the $n^{\mathrm{th}}$ receive antenna of Eve by $g_{m,n}∼\mathrm{CN}\left(0,1\right)$. The received signal of Eve is then written as follows (n,t∈{1,2}):

(17) $$y_{n,t}=g_{n,1}s_{1,t}+g_{n,2}s_{2,t}+w_{n,t},$$

where $w_{n,t}$ is AWGN with the same variance as $v_{n,t}$, i.e., $w_{n,t}∼\mathrm{CN}\left(0,\sigma^{2}\right)$. Suppose that Eve operates optimally for the coherent detection and successive inter-symbol-interference cancellation (SIC) with full CSI (FCSI) including $\left\{h_{n,m}\right\}$ and $\left\{g_{n,m}\right\}$. In other words, we consider the worst case scenario of the secure communications. After the perfect STLC combining and SIC procedure, Eve obtains the estimate of $x_{i}$ as follows:

(18) $$\left[\begin{array}{c} y_{1,1}+y_{2,2}*\\ y_{2,1}-y_{1,2}^{*} \end{array}\right]=\sqrt{1-\alpha }\sqrt{\frac{1}{\gamma }}Ghx_{i}+\sqrt{\alpha }Ga+\left[\begin{array}{c} w_{1,1}+w_{2,2}^{*}\\ w_{2,,1}-w_{1,2}^{*} \end{array}\right],$$

where:

(19a) $$\begin{array}{ccc} G & = & \left[\begin{array}{cccc} g_{1,1} & g_{2,1}^{*} & g_{1,2} & g_{2,2}^{*}\\ g_{2,1} & -g_{1,1}^{*} & g_{2,2} & -g_{1,2}^{*} \end{array}\right], \end{array}$$

(19b) $$\begin{array}{ccc} h & = & {\left[\begin{array}{cccc} h_{1,1}^{*} & h_{2,1} & h_{1,2}^{*} & h_{2,2} \end{array}\right]}^{T}. \end{array}$$

Note that Eve cannot cancel AN even with FCSI. Thus, the effective signal-to-interference-plus-noise ratio (SINR) of Eve is derived from Equation (18) as follows:

(20) $$\rho_{\mathrm{Eve}}=\frac{\left(1-\alpha \right)\epsilon_{h}}{\gamma \left(4\sigma^{2}+\alpha \epsilon_{a}\right)},$$

where the effective Eve channel gain and AN are defined as:

(21) $$\begin{array}{ccc} \epsilon_{h} & ≜ & {∥Gh∥}^{2}, \end{array}$$

(22) $$\begin{array}{ccc} \epsilon_{a} & ≜ & {∥Ga∥}^{2}. \end{array}$$

From Equations (12) and (20), it is clear that the SNR of Bob and the SINR of Eve are the functions of a power loading factor, α. Consequently, for the given channels, the worst-case instantaneous secrecy rate is defined as a function of α as:

(23) $$R\left(\alpha \right)≜{\left[\log_{2}\left(1+\rho_{\mathrm{Bob}}\right)-\log_{2}\left(1+\rho_{\mathrm{Eve}}\right)\right]}^{+},$$

where ${\left[x\right]}^{+}=\max\left(x,0\right)$.

We now design $\alpha^{o}$, which maximizes the worst-case secrecy rate R(α) as follows:

(24) $$\alpha^{o}=\max_{0\leq \alpha \leq 1}{\left[\log_{2}\frac{1+\rho_{\mathrm{Bob}}}{1+\rho_{\mathrm{Eve}}}\right]}^{+}.$$

The equivalent objective function of Equation (24) can be derived as follows:

(25) $$\begin{array}{cc} {\left[\log_{2}\frac{1+\rho_{\mathrm{Bob}}}{1+\rho_{\mathrm{Eve}}}\right]}^{+} & \overset{\left(a\right)}{\equiv }\frac{1+\rho_{\mathrm{Bob}}}{1+\rho_{\mathrm{Eve}}}\\ & =\frac{1+\frac{(1-\alpha )\gamma }{2\sigma^{2}}}{1+\frac{\left(1-\alpha \right)\epsilon_{h}}{\gamma (4\sigma^{2}+\alpha \epsilon_{a})}}\\ & =\frac{\left(4\sigma^{2}+\epsilon_{a}\alpha \right)\left(2\sigma^{2}+\gamma^{2}-\gamma^{2}\alpha \right)}{8\sigma^{4}\gamma +2\sigma^{2}\epsilon_{h}+2\sigma^{2}\left(\epsilon_{a}\gamma -\epsilon_{h}\right)\alpha }\\ & ≜D(\alpha ), \end{array}$$

where (a) comes from the facts that the secure communication is feasible, i.e., $\rho_{\mathrm{Bob}}>\rho_{\mathrm{Eve}}$, and $\log_{2}\left(\cdot \right)$ is a monotonically-increasing function.

Since the convexity of the objective function D(α) in Equation (25) with respect to α depends on the system parameters, namely $\sigma^{2}$, γ, $\epsilon_{h}$, and $\epsilon_{a}$, to find the optimal $\alpha^{o}$, we have to check the critical points including two boundary points 0 and 1. To find the critical point of $\alpha^{\prime }$ between 0 and 1, we relax the feasible reason of α to the entire real values and derive $\alpha^{\prime }$ from the first-order optimality condition, i.e.,

(26) $$\begin{array}{ccc} \frac{\partial D(\alpha )}{\partial \alpha } & = & \frac{\left(4\sigma^{2}+2\sigma^{2}\epsilon_{a}+\epsilon_{a}\right)\gamma^{2}+2\sigma^{2}\epsilon_{a}\gamma }{8\sigma^{2}\gamma +2\sigma^{2}\epsilon_{h}}-\frac{\left(\epsilon_{a}\gamma -\epsilon_{h}\right)\left(4\sigma^{2}+\epsilon_{a}\alpha \right)\left(2\sigma^{2}\gamma +\gamma^{2}-\gamma^{2}\alpha \right)}{{\left(4\sigma^{2}\gamma +\epsilon_{h}+\left(\epsilon_{a}\gamma -\epsilon_{h}\right)\alpha \right)}^{2}}\\ & = & 0. \end{array}$$

By solving Equation (26) with respect to α, we can obtain the critical point $\alpha^{\prime }$ as follows:

(27) $$\alpha^{\prime }=\left\{\begin{array}{cc} \frac{\epsilon_{a}-4\sigma^{2}}{2\epsilon_{a}}+\frac{\sigma^{2}}{\gamma }, & \mathrm{if}\;\gamma =\frac{\epsilon_{h}}{\epsilon_{a}}\\ \frac{\sqrt{\epsilon_{a}\epsilon_{h}c\left(c\gamma^{3}+2\sigma^{2}\left(\epsilon_{a}\gamma -\epsilon_{h}\sigma^{2}\right)\gamma \right)}-\epsilon_{a}\gamma \left(4\sigma^{2}\gamma +\epsilon_{h}\right)}{\epsilon_{a}\left(\epsilon_{a}\gamma -\epsilon_{a}\right)\gamma }, & \mathrm{else}\;\mathrm{if}\;\gamma \geq \gamma_{t}\\ \mathrm{infeasible}, & \mathrm{otherwise}, \end{array}\right.$$

where:

(28a)c=ϵa+4σ2(28b)γt=ϵa2+2ϵaϵh+8σ2ϵh−σ2ϵaϵa+4σ2.

We can then obtain the optimal $\alpha^{o}$ by considering the feasible region of $\alpha^{\prime }$ and two boundaries of α, namely 0 and 1, as follows:

(29) $$\alpha^{o}=\left\{\begin{array}{cc} 0, & \mathrm{if}\;\alpha^{\prime }<0,\\ 1, & \mathrm{if}\;\alpha^{\prime }>1,\\ \alpha^{\prime }, & \mathrm{otherwise} \end{array}\right.$$

From Equation (29), the interesting remarks, which can be a guideline to design the AN power loading factor, are obtained as follows:

**Remark** **1.** *As the channel quality between Alice and Bob becomes better, i.e., γ increases, more power is allocated to the intended signals, i.e., α decreases, to further exploit the good quality of the main channels.*

**Remark** **2.** *As the quality of the eavesdropper channel between Eve and Alice becomes better, i.e., $\epsilon_{a}$ and $\epsilon_{h}$ increase, more power is allocated to the AN signals, i.e., α increases, to hinder Eve from eavesdropping.*

**Figure 2:**
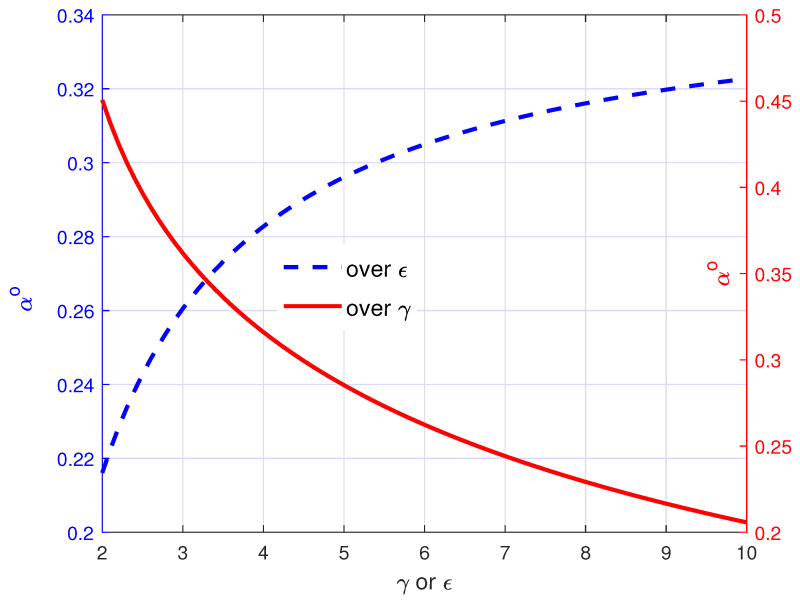
Optimal power loading factor $\alpha^{o}$ for artificial noise (AN) over γ when $\epsilon_{a}=\epsilon_{h}=\epsilon =8$ and $\sigma^{2}=0.1$ and over ϵ when γ=4 and $\sigma^{2}=0.1$.

The remarks are intuitively reasonable. If Eve’s channel is dominant, more power is allocated to AN as it is more efficient for the secrecy rate improvement to reduce Eve’s rate, i.e., the second term inside the brackets in Equation (23). On the other hand, if Bob’s channel is dominant, more power is allocated to the intended signals as it is more efficient for the secrecy rate improvement to increase Bob’s rate, i.e., the first term inside the brackets in Equation (23). The remarks are verified through the simulation results in Figure 2. Figure 2 shows the optimal power loading factor $\alpha^{o}$ for AN, according to γ and ϵ, where we set $\epsilon_{a}=\epsilon_{h}=\epsilon$ and $\sigma^{2}=0.1$. For varying γ, we set ϵ=8, while for varying ϵ, we set γ=4. From these results, we clearly observe that the optimal AN power increased as ϵ increased or as γ decreased, as stated in Remarks 1 and 2.

**Figure 3:**
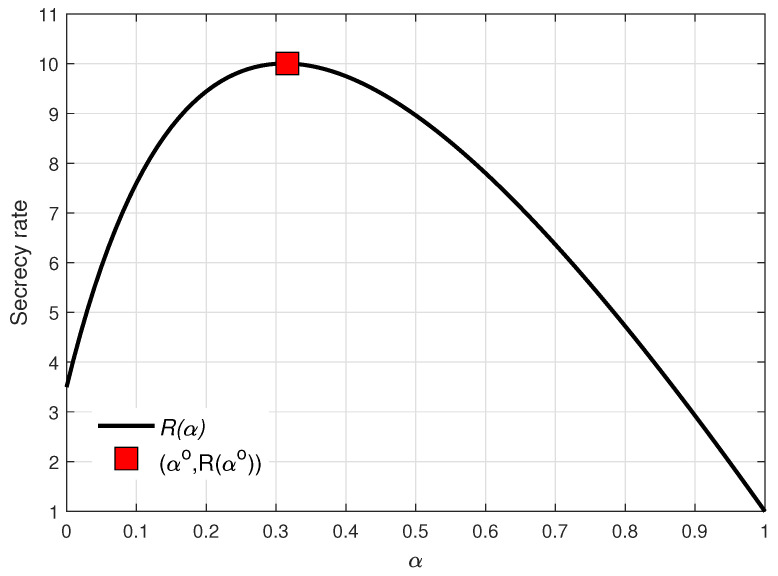
Secrecy rate R(α) over α when ϵ=8, γ=4, and $\sigma^{2}=0.1$.

Numerical results verifying the optimality of $\alpha^{o}$ in Equation (29) are shown in Figure 3. As observed in Figure 3, $\alpha^{o}$ achieved the maximum of the equivalent cost function D(α) in Equation (25) in the feasible region; as a result, the designed $\alpha^{o}$ in Equation (29) provided the maximum secrecy rate.

## 4. Numerical Results

**Figure 4:**
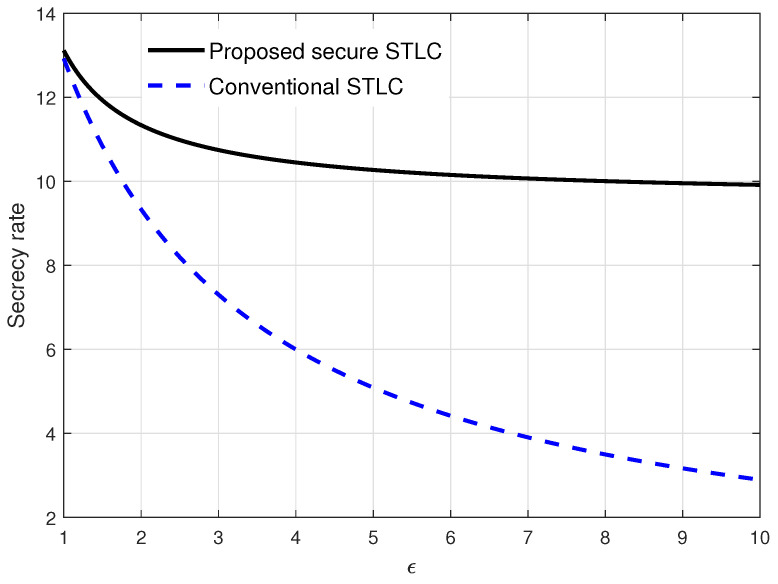
Secrecy rate evaluation results over Eve’s channel quality ϵ when γ=4 and $\sigma^{2}=0.1$.

In this section, we compare the proposed secure STLC with AN and the conventional STLC without AN. In Figure 4, the secrecy rate in Equation (23) has been evaluated over Eve’s channel quality ϵ when γ=4 and $\sigma^{2}=0.1$. Obviously, the secrecy rate decreased as ϵ increased. However, the secrecy rate’s decrease of the speed of the proposed secure STLC was relatively moderate compared to that of the conventional STLC. From the results, we see that the proposed secure STLC always outperformed the conventional STLC; especially, Eve’s channel gain was relatively stronger than Bob’s channel gain.

**Figure 5:**
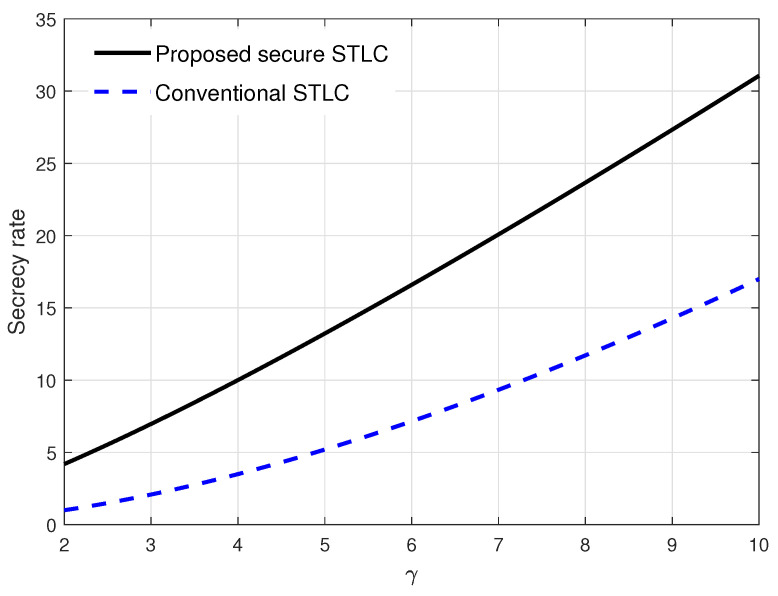
Secrecy rate evaluation results over Bob’s channel quality γ when ϵ=8 and $\sigma^{2}=0.1$.

In Figure 5, the secrecy rate in Equation (23) was evaluated over Bob’s channel quality γ when ϵ=8 and $\sigma^{2}=0.1$. Obviously, the secrecy rate increased as γ increased. Here, the secrecy rate’s increase of the speed of the proposed secure STLC was relatively faster compared to the conventional STLC. From the results, we see that the proposed secure STLC always outperformed the conventional STLC; especially, Bob’s channel gain was relatively stronger than Bob’s channel gain.

From the results in Figure 4 and Figure 5, it is verified that the proposed secure STLC system with AN always outperformed the conventional STLC system, i.e., $R\left(\alpha^{o}\right)>R\left(0\right)$. Especially, when the main and eavesdropper channels were highly asymmetric, i.e., one channel gain was relatively stronger than the other one, the proposed secure STLC system could significantly improve the secrecy rate compared to the conventional STLC system.

**Figure 6:**
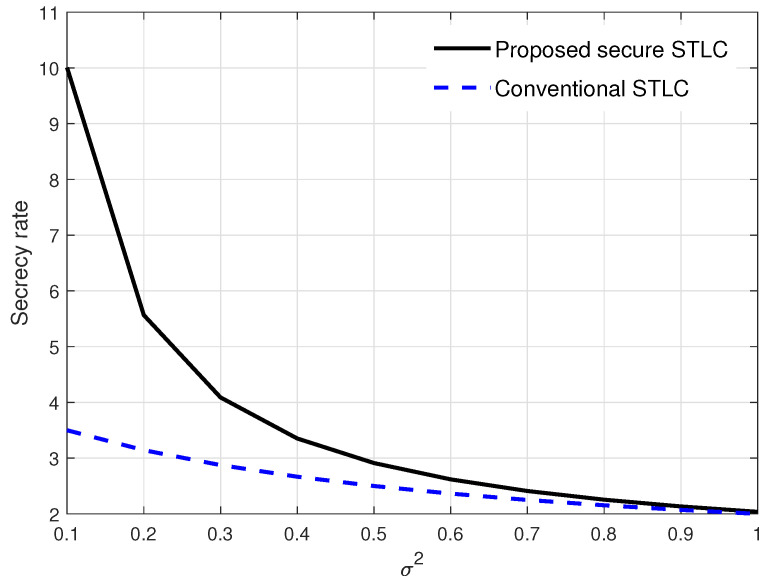
Secrecy rate evaluation results over noise power when γ=4 and ϵ=8.

In Figure 6, the secrecy rate was evaluated over noise variance $\sigma^{2}$. As expected, the secrecy rate decreased as $\sigma^{2}$ increased, and both secrecy rates of the proposed secure STLC and the conventional STLC were merged at the low secrecy rate as both Bob and Eve achieved very low rates. From the results, we see that the proposed AN with power control played a crucial role in the secure communication, especially in the high system SNR regime.

## 5. Conclusions

In this paper, we have designed artificial noise and its optimal power loading factor to improve the secrecy rate of a 2×2 space-time line code (STLC) system. The proposed secure STLC systems can significantly improve the secrecy rate of the conventional STLC system, as verified by the numerical results.
